# Probabilistic Risk Assessment of Polycyclic Aromatic Hydrocarbons in a Colombian Reservoir

**DOI:** 10.1007/s00128-022-03567-7

**Published:** 2022-07-23

**Authors:** F. Amaringo, Y. Puerta, F. Molina

**Affiliations:** 1 Research Group in Management and Environmental Modeling, GAIA, University of Antioquia, Medellín, Colombia; 2 Research Group GeoLimna, Faculty of Engineering, University of Antioquia, 67th Street # 53 - 108, Medellín, Colombia

**Keywords:** PAHs, Semipermeable membrane devices, Source, Pyrogenic, Petrogenic, Risk assessment

## Abstract

The purpose of this research was to evaluate the concentrations, sources and ecological risk assessment of sixteen polycyclic aromatic hydrocarbons (PAHs) present in water from the La Fe reservoir, Colombia in the months of October and November of 2017 and 2018. Concentrations of PAHs in water were measured with semipermeable membrane devices (SPMD) which allow obtaining the dissolved concentrations of the PAHs in the reservoir, emphasizing the reactivity and bioavailability in the environment. The PAHs analyses were carried out by means of gas chromatography, coupled with tandem mass spectrometry (GC/MS–MS) with triple quadrupole (QqQ). The environmental risk assessment using the estimation of risk quotient with deterministic and probabilistic method, the predictive no-effect concentration (PNEC) and environmental exposure concentration (EEC) in water indicate a negligibe risk for probabilistic method for all PAHs evaluated (RQ < 0.1).

**Supplementary Information:**

The online version contains supplementary material available at 10.1007/s00128-022-03567-7.

Polycyclic aromatic hydrocarbons (PAH) are a type of organic compound that has two or more fused benzene rings within its chemical structure (Ehrenhauser 2015), which convert them into non-polar, hydrophobic, highly toxic, highly stable substances in the environment with great resistance to microbial degradation (Nikitha et al. 2017).These compounds can be transported over a long range of distances and enter the water through air–water exchange (Fang et al. 2012), forming suspended particles that are then easily deposited in aquatic sediments due to their hydrophobicity, which causes adsorption in surface sediments (Soliman et al. 2014). PAHs not only affect human health, through the ingestion of water and contact with the skin, but also sensitive species of different taxonomic groups in the aquatic environment (Sun et al. 2015). From these species, dose–response assessment can be developed, and therefore, daily exposure and risk indexes of these organic pollutants can be calculated. These estimates are key to manage the reduction of the emission and impact if the risk levels are harmful to the environment (Soltani et al. 2015). One of the newest methods for sampling trace pollutants in water are semipermeable membrane devices (SPMD) which can be used as in-situ monitors of dissolved pollutant concentrations and permit risk assessment exercises to evaluate the potential toxicity of pollutant mixtures, which is not possible with direct sampling methods (Petty et al. 2000). SPMD allow a passive sampling in situ, containing in their interior triolein that simulates processes of bioaccumulation in fish, and allowing the analysis of persistent organic pollutants (POPs) or other hydrophobic organic pollutants with the octanol/water partition coefficient greater than 3 (log *Kow* ≥ 3) as Polycyclic aromatic hydrocarbons (PAH), polychlorinated biphenyls (PCB), and organochlorine pesticides (Huckins et al. 2006; Narvaez and Molina 2012). The purpose of this study was to evaluate the presence, sources and environmental risk associated with PAHs found in La Fe reservoir. This study is essential to understand the risk faced by species and people who make use of this water body. The results contribute to the monitoring of PAH to determine the transport and destination of pollutants and mitigate the impact of these organic compounds on the environment using the risk quotient determined with probabilistic approach.

## Materials and Methods

**Fig. 1:**
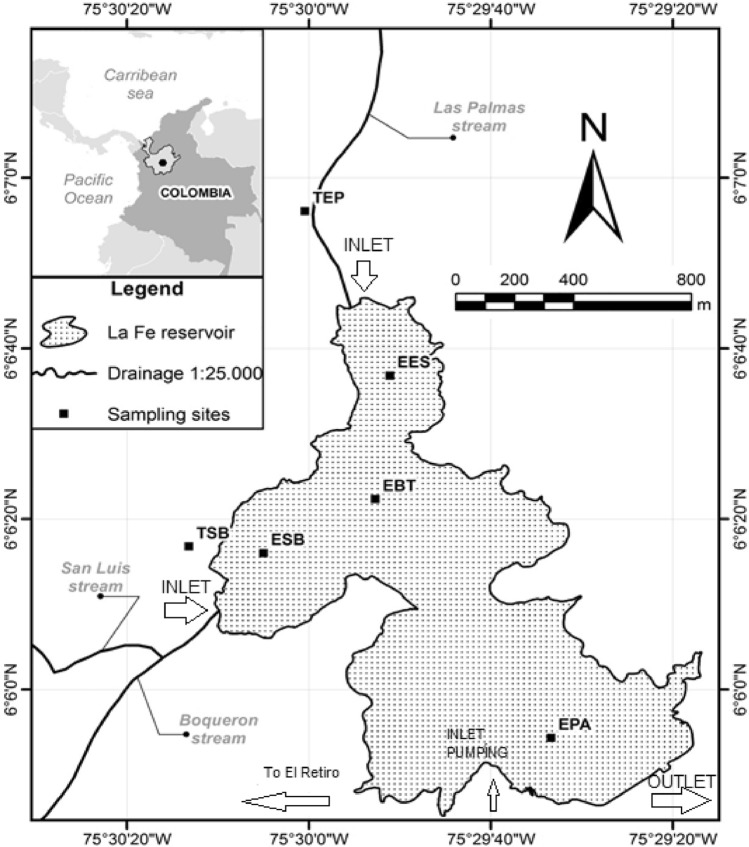
Map of La Fe reservoir with the sampling stations San Luis-Boquerón tributary (TSB), Palmas-Espiritu Santo tributary (TPE), Espiritu Santo Inlet (EES), Catchment tower outlet (EBT), San Luis – Boquerón Inlet (ESB) and Pantanillo River Inlet (EPA)

La Fe reservoir is located in the municipality of El Retiro, Antioquia (NW Colombia) (Fig. 1). The reservoir has a volume of 15 mm^3^, an area of 1.33 km^2^, an altitude of 2155 m above sea level, and is surrounded by a high traffic road that leads to the eastern municipalities Antioquia. This reservoir supplies water for consumption of two million people in the Metropolitan Area of the Aburra Valley. A pumping system from the Pantanillo River is used to regulate the level of the reservoir, which supplies the metropolitan aqueduct of about one million inhabitants in Medellin City through the Ayurá water potabilization plant (Hernani and Ramirez 2002). Four sampling campaigns were conducted in the months of October and November of 2017 and 2018 to compare the results after one year in six stations: San Luis-Boquerón tributary (TSB), Palmas-Espiritu Santo tributary (TPE), Espiritu Santo Inlet (EES), Catchment tower outlet (EBT), San Luis – Boquerón Inlet (ESB) and Pantanillo River Inlet (EPA). The first two stations are located at the entrance of the tributaries and the rest are located within the reservoir (Fig. 1). Water samples were collected by suspending stainless steel baskets 2 m below water surface at each station, where three SPMD were placed and retrieved after 21 day of exposure. From each station, an extract was obtained (24 extracts in total for all stations).

The SPMDs were obtained from Environmental Sampling Technologies EST-Lab, St. Joseph, MO, USA, with the following specifications: 99% triolein, LDPE of 450 cm^2^ and thickness of 75 µm. The procedure for the determination of PAH in SPMD was in agreement with Pogorzelec and Piekarska (2018). The collected membranes were first washed with a brush to remove biofilm and residues, then stored in aluminum foil and finally transported to the laboratory in sealed bags to avoid cross-contamination. In the laboratory, the membranes were washed with a 10% HCl solution to remove the inorganic salts and the biofilm adhering to the surface, followed by a wash with deionized water and isopropanol to remove the water residues. To separate the triolein from the organic compounds, the SPMDs were dialyzed in 500 mL Erlenmeyer flasks containing 200 mL of n-hexane.

After 24 h of dialysis, the solvent was replaced and dialyzed again for 10 h. The triolein was separated with a Bio-beads SX-3 200–400 mesh column, by gel permeation chromatograhy (GPC) (Klučárová et al. 2013) with dichloromethane. A cleanup was done with acetone-hexane by a column with silica-activated gel. The extract was concentrated by means of nitrogen flow.

The concentration of the 16 priority PAHs – Benzo[a]pyrene (BaP), Benzo[b]fluroanthene (BbF), Benzo[g,h,i]periylene (BghiP), Benzo[k]fluoranthene (BkF), Benzo[a]anthracene (BaA), Chrysene (Chr), Dibenzo(a,h)anthracene (DahA), Fluoranthene (Flu), Fluorene (F), Indeno(1,2,3-cd)pyrene (Ind), Phenanthrene (Phe), Pyrene (Pyr) Acenaphthene (Ace), Acenaphthylene (Acy), Anthracene (Ant) and Naphthalene (N) – named by the EPA (Achten and Andersson 2015) was determined by Gas Chromatograph (GC) (Bruker 451), equipped with a PTV Injector for capillary columns, coupled to a Triple mass spectrometer (Bruker SCION TQ) with triple quadrupole analyzer (QqQ) and electronic Impact Ionization (EI) source. For the GC analysis, the external standard column BR5, splitless mode for PAH in MRM mode was used, with EPA PAH Mix (2000 μg/mL) in dichloromethane. Each calibration point was prepared for triplicate. Calibration curve were performed with R^2^ between 0.9904 and 0.9984, LOD between 1.08–9.76 ng/mL and LOQ between 3.62–30.28 ng/L. The recovery percentages were determined with the PAH Mix reference material and the values vary between 80% and 120% (Table S1 and S2). Oven temperature was initially set at 110°C, and increased to 310°C at a rate of 10°C (3.5 min hold time).

Performance reference compounds (PRC) are used to evaluate the SPMD-water exchange kinetics in situ, which are added to the membrane before exposure. The chosen PRCs cannot be present in the environment, normally Polychlorinated Biphenyls (PCB) 14, 29 and 50 are chosen.

The dissipation of the PRC is equal to Eq. (1),

1 $$N_{PRC} = N_{0PRC } {\text{exp}}( - k_{e} t)]$$

where, $N_{PRC}$ is the amount of PRC in the SPMD membranes and $N_{0PRC}$ is the amount of PRC at t = 0. By measuring $N_{PRC} /N_{OPRC}$ we can estimate the elimination constant *(ke)* using Eq. (2).

2 $$ke = - ln (N_{PRC} /N_{0PRC)} )/t$$

When, $k_{e} t$ <  < 1, $C_{SPMD}$ is equal to Eq. (3),

3 $$C_{SPMD} = K_{sw } C_{w} k_{e} t$$

Because $C_{SPMD}$ increases linearly with time, this phase is called "linear absorption model" or "kinetic sampling". And the sampling is integrated with time, this type of model occurs when there are short periods of exposure or highly hydrophobic compounds.

Therefore, the amount of analyte absorbed (N) by the SPMD membranes during kinetic sampling is equal to Eq. 4, where $V_{SPMD}$ is the volume of the SPMD membrane.

4 $${\text{N}} = V_{SPMD} K_{sw } C_{w} k_{e} t$$

From Eq. 4, we define the sampling rate (*Rs*) for kinetic sampling, using Eq. 5,

5 $$Rs = V_{SPMD} K_{sw } k_{e}$$

The sampling rate (*Rs*) provides a conceptual framework between classical batch extraction techniques and passive SPMD sampling, since *Rst* is equal to the volume of water extracted (Huckins et al. 2006).

A MANOVA test was initially conducted to compare the concentrations of the hydrocarbons at the different sampling stations. However, due to lack of compliance in the assumptions, a Kruskal–Wallis test was applied and finally a one-way ANOVA for the hydrocarbon Ant, in order to identify differentiated concentrations of this compound among the stations. For the ANOVA, a residual analysis was carried out to verify the assumptions of normality (Shapiro–Wilk test), homoscedasticity (Levene test) and independence (Autocorrelation function (acf)). The Tukey’s post hoc test was to contrast pairwise differences among the locations. All analyzes were performed in statistical software R (R Core Team 2018).

To determine the possible sources of PAH in the dialyzed extracts of SPMD, the ratios of PAH’s molecular indices were used. The most common isomer ratios are Flu/(Flu + Pyr), Ant/(Phe + Ant), BaA/(BaA + Chr), Ind/(Ind + BghiP) (Gdara et al. 2017), Fen/Ant, Flu/Pyr and BaP/BghiP. If the Flu/(Flu + Pyr) ratio is < 0.4, the origin is petrogenic; between 0.4 and 0.5, the origin is a mixture of petrogenic and pyrogenic sources, whereas if it is greater than 0.5, the origin is pyrogenic. If Ant/(Phe + Ant) is < 0.1, the origin is petrogenic, if it is > 0.1, the origin is pyrogenic. If the ratio of Ind/Ind + BghiP is < 0.5, the origin is pyrogenic while values greater than 0.5, the sources are of petrogenic origin (Yilmaz et al. 2014).

The ecological risk assessment (ERA) is a tool used in the organization, structuring and collection of scientific data, that allows the identification of potential dangers in order to establish priorities in regulatory control and apply corrective actions (Chen and Liu 2014). ERA is performed by obtaining Predictive No-Effect Concentration (PNEC) (Wu et al. 2011), which can be calculated from deterministic and probabilistic approaches. The deterministic approach uses the lowest value of acute (LC_50_) or chronic (NOEC) toxicity in relation to an Assessment Factor (AF) for each PAH. The probabilistic method applies the extrapolation of a set of toxicity data in different taxonomic groups of at least 15 species that allow the construction of a species sensitivity curve (SSD), from which the PNECp value is calculated (Puerta et al. 2019; EC 2011).

The ETX 2.1 software allows the calculation of the SSD curve for each of the compounds (Fig. 4). To guarantee data quality, the Anderson–Darling, Kolgomorov-Smirnov and Cramer Von Mises normality tests were performed, which are used as criteria for the parametric distribution of the data all levels (0.1, 0.05, 0.025, and 0.01). The figures of the species sensitivity curves were made with the RStudio software version 4.0.1.

## Results and Discussion

**Fig. 2:**
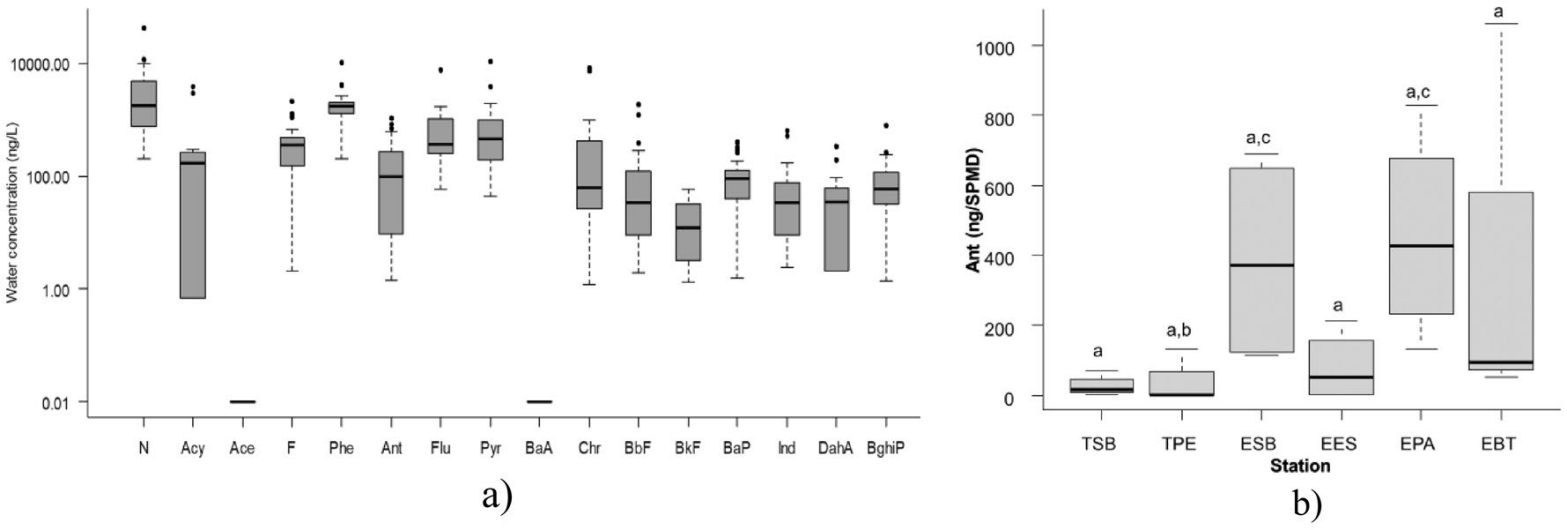
**a** Boxplot for the estimated exposure concentration of the 16 PAHs dissolved in the La Fe reservoir water. **b** Boxplot for Anthracene (Ant) in sampling stations. The different letters indicate a significant difference in Ant concentrations in relation to the sampling stations (*p* < 0.05)

Of the 16 parent PAHs analyzed, 14 were detected, and only two (Ace and BaA) were not found in the range of detection of the equipment. The most abundant PAHs were N, Pyr, Flu and Phe, F and Chr (Fig. 2a). The average concentration of PAH of four samples with SPMD in six stations of the water reservoir were in a range of ∑PAHs 5.762 ng/g.SPMD in the TSB station, up to a maximum of 21.491 ng/g.SPMD at the EBT station, being the average 11.447 ng/g.SPMD (Table 1).

**Table 1:** Concentrations of PAH (C_SPMD_) in the reservoir stations (ng/g.SPMD) (Mean ± Standard error) and molecular index of PAH

PAH	TSB	TPE	EES	EBT	ESB	EPA
N	3845 ± 2707	3932 ± 2147	3896 ± 2628	12,062 ± 10,365	2337 ± 1099	2549 ± 1093
Acy	73.9 ± 61.6	161.8 ± 57.5	892.4 ± 702.8	69.0 ± 68.3	1069 ± 933	107.9 ± 63.6
Ace	–	–	–	–	–	–
F	148.7 ± 88.9	239.9 ± 85.5	460.2 ± 236.6	941.7 ± 404.7	267.5 ± 55.4	601.6 ± 246.8
Phe	812.8 ± 376.5	1335 ± 159.4	2308 ± 669	1928 ± 75.5	2386 ± 664	3965 ± 2168
Ant	26.7 ± 14.7	34.1 ± 32.6	386.1 ± 152.8	453.8 ± 148.8	79.1 ± 50.3	325.5 ± 245.3
Flu	317.9 ± 161.6	373.8 ± 136.5	842.8 ± 333.3	1307 ± 251.3	557.7 ± 197.8	2208 ± 1797
Pyr	235.9 ± 121.4	365.8 ± 184.8	815.8 ± 350.1	1684 ± 760.9	1017 ± 357.9	3016 ± 2641
BaA	–	–	–	–	–	–
Chr	60.3 ± 46.2	148.5 ± 118.7	236.7 ± 128.8	2225 ± 1767	212.1 ± 196.9	2167 ± 2063
BbF	23.3 ± 15.2	38.6 ± 29.4	58.2 ± 36.2	438.9 ± 263.8	100.9 ± 66.0	500.8 ± 450.4
BkF	13.8 ± 6.6	21.3 ± 9.3	22.4 ± 13.2	9.07 ± 7.75	31.7 ± 11.7	22.58 ± 7.85
BaP	81.6 ± 25.8	95.5 ± 9.4	38.8 ± 26.2	166.7 ± 47.6	169.6 ± 74.6	132.0 ± 91.0
Ind	24.5 ± 14.9	27.0 ± 10.1	56.7 ± 28.2	218.0 ± 147.7	58.1 ± 38.4	167.8 ± 116.9
DahA	46.17 ± 9.9	41.7 ± 15.5	–	86.7 ± 84.5	29.2 ± 16.8	83.7 ± 42.2
BghiP	51.1 ± 19.8	62.6 ± 26.9	60.4 ± 19.5	168.8 ± 47.2	77.9 ± 24.9	235.4 ± 185.4
∑PAH	5762	6878	10,077	21,491	8393	16,082
Flu/(Flu + Pyr)	0.57	0.51	0.51	0.38	0.35	0.42
Ant/(Fen + Ant)	0.03	0.02	0.14	0.19	0.03	0.08
Flu/Pyr	1.35	1.02	1.03	0.62	0.55	0.73
Phe/Ant	30.45	39.16	5.98	4.25	30.15	12.18
Ind/Ind + BghiP	0.32	0.3	0.48	0.56	0.43	0.42
BaP/BghiP	1.6	1.53	0.64	0.99	2.18	0.56

The Kruskal–Wallis for all the PAHs shows statistically significant differences between the concentration of Anthracene and the sampling stations. The ANOVA for anthracene presents a significant difference (*p* < 0.05) between the ESB-TPE stations (*p* = 0.035) and EPA-TPE stations (*p* = 0.022) (Fig. 2b).

The concentrations of PAH in the six sampling stations during the two years ranged from ∑PAHs 219.28 ng/L in the TPE station and 1.002 ng/L at the EBT, being the average 420 ng/L for all stations (Table S3 and S4). The PAHs with higher estimated concentrations (*Cw*) are those with low molecular weight such as N, Acy, Phe, Flu, and Pyr which have 2 to 4 fused benzene rings in their chemical structure. High molecular weight hydrocarbons with 5 to 6 fused rings present low concentrations between 0.012 and 0.67 ng/L, among them BaP (0.19 ng/L). These PAH are considered carcinogenic by the World Health Organization (WHO), which established an upper limit of 50 ng/L for their presence in water (WHO 1998).

**Fig. 3:**
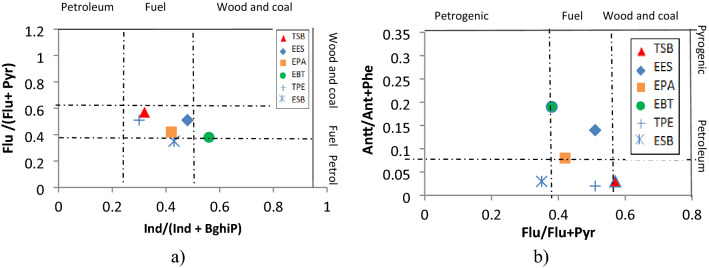
**a** Cross plot for the sources of PAH, Flu /(Flu + Pyr) vs. Ind/(Ind + BghiP) in six sampling stations. **b** Cross plot for the sources of PAH Flu /(Flu + Pyr) vs. Ant/(Ant + Phe) in six sampling stations

Table 1 shows the values of molecular indices that allow the determination of the petrogenic or pyrogenic sources of PAHs. Figure 3a and b show the cross plots for Ind/(Ind + BghiP) vs. Flu/(Flu + Pyr) and Ant/(Ant + Phe) vs. Flu/(Flu + Pyr), which allow determining if the sources come from petroleum fuel or from coal and wood.

**Fig. 4:**
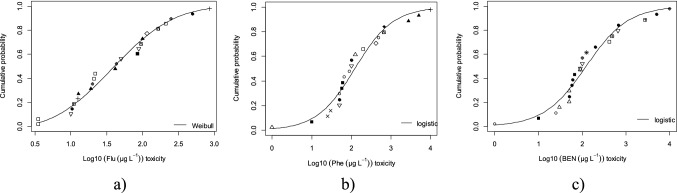
Sensitive species distributions for **a** Fluoranthene, **b** Phenantrene and** c** Benzo(a)pyrene ◊ = Annelida; ○ = Marine-Fish; ● = Fish; ▲ = Microalgae; □ = Marine-zooplankton; ■ = zooplankton; **╳** = Marine-amphipoda; △ = Marine-microalgae; ▽ = Mollusca; ⊞ = Frog;  = Insect; ◆ = Marine-annelida; +  = Amphipoda; = Macrophyte;  = Reptile;  = Protozoan

Table 2 shows the aquatic PNEC for the three PAHs, obtaining values of 4.21, 3.66 and 0.029 µg/L for Phe, Flu and BaP respectively. These values were calculated from the chronic toxicity data for different reported species in the literature. Figure 4 shows the sensitivity curves of the SSD species for each compound analyzed.

**Table 2:** PNEC probabilistic, mean, S.D and n for Phenantrene, Fluoranthene and Benzo(a)pyrene

Parameters	Phenantrene	Fluoranthene	Benzo(a)pyrene
Mean	2.13	1.63	0.64
S.D	0.90	0.64	1.30
n	22	24	26
PNEC	4.21	3.6	0.029

**Fig. 5:**
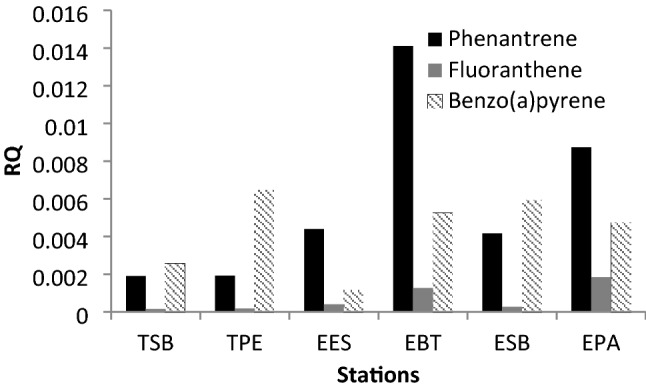
Risk quotient for Phenantrene, Fluoranthene and Benzo(a)pyrene

The EEC values for the three PAHs are those reported in Table S4. By replacing these values and those of the PNECp. Figure 5 shows the RQs for all sampling stations. RQ < 0.1 indicate negligible ecological risk of these PAHs in the reservoir, which occurs for the three PAHs. Aquatic acute toxicity data evaluated in different ecological groups for Phenanthrene, Fluoranthene, and Benzo(a)pyrene are shown in Tables S6, S7 and S8 respectively.

The results obtained for both the n-hexane solvent and the blanks indicate no contamination during the deployment and treatment of the membranes. The PAH values found in the SPMD extracts of the TSB and TPE tributaries are lower than those found in the stations within the reservoir, possibly due to the longer residence time of the water in the reservoir, which allows a greater diffusion of the water towards membranes and therefore an accumulation in triolein (Prest and Jacobson 1997). To highlight the importance of the use of passive sampling compared to traditional sampling, the results of this study can be compared with those of (Serna 2012) where they analyzed the 16 PAHs in La Fe reservoir stations and could not report any because they were below the quantification limit of the gas chromatography equipment.

The PAH concentrations in the TPE and EPA stations were the highest, possibly due to the fact that TPE collects the water of the reservoir in the outlet to the La Ayura Potabilization Plant. As for the EPA station, the high concentrations may be due to the strong industrial and mining activities from the Pantanillo River and the Buey Stream in El Retiro (Salazar 2017).

In this study, none of the samples exceeded the regulatory guidelines for water quality established by the World Health Organization (WHO 1998), which shows that the water from La Fe reservoir can be used for potabilization and human consumption since the concentrations are very low. These results are similar to those reported in the Three Gorges Reservoir in China, where 6 sites were analyzed during 7 and 24 days, where the highest values were obtained for Phenanthrene, Fluorene, Chrysen, Pyrene and Naphthalene (Wang et al. 2009), as well as the study of PAHs in 6 rivers and creeks of the Milwaukee Metropolitan Sewerage District area of Wisconsin in 37 days of passive sampling of July–August, 2007, where 35 and 560 ng/L of total PAHs were found (USGS 2014).

From Table 1, the values of the ratio of Flu/(Flu + Pyr) isomers shows that the origin of the PAHs dissolved in water are of pyrogenic origin in the TSB, TPE, EES stations and a mix between petrogenic and pyrogenic origins in the EBT and EPA stations. The values of the Ind/(Ind + BghiP) isomer ratio show that most of the stations are of pyrogenic origin, with values < 0.5, except for the EBT station that has a value > 0.5, indicating petrogenic origin. The Ant/(Phe + Ant) ratio shows that three stations (EPA, EBT, EES) are of pyrogenic origin since they have values < 0.1, whereas the other stations (TSB, TPE, ESB) are of petrogenic origin, with values > 0.1. Regarding the BaP/BghiP ratio, the source of most of the stations is of pyrogenic origin, since the ratio is > 0.6 due to vehicular emissions, probably due to the proximity to the highway that borders the reservoir, with the exception of the EES station, with a value less < 0.6, indicating sources other than vehicular emissions. According to the residues of the molecular index, most of the stations present PAH of pyrogenic origin, probably due to the incomplete combustion of diesel and gasoline engines, forest fires or the combustion of coal or residues.

The cross plots for Ind/(Ind + BghiP) vs. Flu/(Flu + Pyr) and Ant/(Ant + Phe) vs. Flu/(Flu + Pyr) serve to establish that the origin of the PAH in most of the stations is fuel, derived from the incomplete combustion of automobiles that circulate around the reservoir and are transported to the reservoir by means of the winds (Chapman 1996). In the same way, Fig. 3 shows the cross plot of Ant/(Ant + Phe) vs. Flu/(Flu + Pyr), which establishes the pyrogenic origin of the PAHs at all sampling stations in the reservoir.

On the other hand, Fig. 3a and b allowed us to establish that PAHs sources in most of the different stations originate from fuel (58.3%), while 20% come from a fuel–oil mixture and 16% from a fuel-combustion mixture of coal and firewood, indicating that 75% of the stations have a pyrogenic origin.

The sensitivity curve of the species for the three compounds analyzed present different ecological groups as the most sensitive: the marine zooplankton (ZP-MAR) and mollusca (MOL) For Flu; marine fish (FISH MAR) and zooplankton (ZP) for BaP; and marine microalgae (MIC-MAR) and zooplankton (ZP) for Phe (Fig. 4). The previous ecological groups are the most sensitive to each hydrocarbon. Based on the response of these ecological groups, the protection of the environment against contamination with these three compounds should be managed. The results of the probabilistic PNEC of the PAHs Phe and BaP found in our research with values of 4.21 and 0.029 (Table 2) are approximately double compared to the work of Wang et al. (2014), who calculated the values of the probabilistic PNEC for Phe and BaP in 2.33 and 0.011 µg/L. The RQ values calculated from the probabilistic PNEC for Phe, Flu and BaP indicate that there is no environmental risk due to the presence of these three hydrocarbons (Fig. 5). The PAHs evaluated by the probabilistic approach present low ecological risk, since they show risk ratios < 0.1 in all the stations. The PAH Phenanthrene is the one that exhibits the highest RQ values in the EPA and EBT stations with 0.0087 and 0.014 respectively (Zheng et al. 2016).

Fourteen PAHs were detected in the SPMD matrix at the six stations in La Fe reservoir and tributaries during the two-year sampling. Sediments presented higher concentrations than water, possibly due to the fact that PAHs, being organic compounds, have low solubilities and high octanol/water partition coefficient (*log Kow*) and due to the short periods of residence of the water in the tributaries and in the reservoir. The SPMD is a reliable and effective tool for the evaluation of contaminants in water, since it allows the estimation of the PAH concentration.

The analysis of molecular indices of isomers of PAH, allowed to determine the pyrogenic origin of the PAH in the sampling stations. On the other hand, the probabilistic approach of the distribution of sensitive species (SSD) of three predominant PAHs in the reservoir (Fen, Flu and BaP), established that there is no risk (RQ < 0.1). This approach has advantages over as long as the reliability of the toxicity values used for the construction of the curve is guaranteed. Therefore, the evaluation of the ecological risk carried out through the deterministic and probabilistic approaches, allowed to establish that the ecosystem is not vulnerable to the vast majority of the 16 PAH evaluated.

## Supplementary Information

Below is the link to the electronic supplementary material.

Supplementary file1 (DOCX 60 kb)
