# Supplementary material for: Probabilistic Risk Assessment of Polycyclic Aromatic Hydrocarbons in a Colombian Reservoir
Source: Bull Environ Contam Toxicol. 2022 Jul 23;109(3):518–25. doi: 10.1007/s00128-022-03567-7 (PMC9392700; doi:10.1007/s00128-022-03567-7)
Supplement: Supplementary file 1 — Supplementary file1 (DOCX 60 kb) [file 128_2022_3567_MOESM1_ESM.docx]

**SUPPLEMENTARY MATERIAL**

**ECOLOGICAL RISK ASSESSMENT OF POLYCYCLIC AROMATIC HYDROCARBONS IN WATER OF LA FE RESERVOIR, COLOMBIA**

***Amaringo, F^1^; Puerta, Y^2^; Molina, F^1^**

^1^*Research Group in Management and Environmental Modeling, GAIA, University of Antioquia, Medellin, Colombia.*

^2^ *Research Group GeoLimna, Faculty of engineering, University of Antioquia, Medellin, 67th street # 53 - 108, Colombia.*

Table S1. Retention times, RSD, correlation coefficients and equation to determine linearity

| HAP | t_R_(min) | RSD(%) | R^2^ | Equation |
| --- | --- | --- | --- | --- |
| N | 5.048 | 41.82 | 0.9984 | Y=1.381x10^4^ X + 4605.3 |
| Acy | 7.775 | 10.46 | 0.9948 | Y=2.891x10^4^ X + 67.27 |
| Ace | 8.073 | 18.55 | 0.9964 | Y=4.019x10^4^ X + 5506.7 |
| F | 8.955 | 17.54 | 0.9925 | Y=2.265x10^4^ X + 1752.3 |
| Fen | 10.754 | 48.27 | 0.9954 | Y=1.483x10^4^ X + 6285.6 |
| Ant | 10.856 | 8.049 | 0.9945 | Y=1.259x10^4^ X - 189.32 |
| Flu | 13.266 | 13.91 | 0.9912 | Y=2.676x10^4^ X + 530.27 |
| Pir | 13.755 | 13.90 | 0.9905 | Y=2.760x10^4^ X + 362.74 |
| BaA | 16.520 | 8.532 | 0.9940 | Y=1.564x10^4^ X + 367.95 |
| Cri | 16.594 | 9.663 | 0.9904 | Y=2.255*x*10^4^ X -931.30 |
| BbF | 18.886 | 7.979 | 0.9956 | Y=1.208x10^4^ X + 334.94 |
| BkF | 18.939 | 10.15 | 0.9937 | Y=1.715x10^4^ X – 1089.2 |
| BaP | 19.541 | 6.498 | 0.9943 | Y=1.046x10^4^ X - 468.90 |
| Ind | 21.931 | 8.491 | 0.9974 | Y=7.895x10^3^X + 215.20 |
| DahA | 22.003 | 13.39 | 0.9942 | Y=1.017x10^4^ X - 900.74 |
| BghiP | 22.585 | 5.606 | 0.9966 | Y=1.187x10^4^ X + 470.26 |

Table S2. Limits of detection LOD and quantification LOQ

|  | LOD (3σ/s) | LOQ(10σ/s) | Quan Ions |
| --- | --- | --- | --- |
| N | 9.08 | 30.28 | 78,102 |
| Acy | 1.08 | 3.62 | 126,151 |
| Ace | 1.38 | 4.62 | 152,127 |
| F | 2.32 | 7.74 | 163,164 |
| Fen | 9.76 | 32.54 | 177,176 |
| Ant | 1.92 | 6.39 | 128.5,152 |
| Flu | 1.56 | 5.19 | 152,201 |
| Pir | 1.51 | 5.03 | 201,200 |
| BaA | 1.63 | 5.45 | 226,202 |
| Cri | 1.28 | 4.28 | 202,226 |
| BbF | 1.98 | 6.60 | 248,250 |
| BkF | 1.77 | 5.92 | 248,250 |
| BaP | 1.86 | 6.21 | 224,250 |
| Ind | 3.23 | 10.75 | 272,274 |
| DahA | 3.95 | 13.16 | 250,276 |
| BghiP | 1.42 | 4.72 | 272,274 |

Table S3. Values of *log Kow, Log Ksw, Log ai y Rs* of the calibration of the SPMDs

| PAH | *Log Kow* | *log Ksw,i* | *log ai* | *Rs,i* |
| --- | --- | --- | --- | --- |
|  | | (mL/mL) |  | (L /d) |
| N | 3.45 | 3.47 | 4.5 | 38.91 |
| Acy | 4.08 | 4.17 | 4.76 | 70.4 |
| Ace | 4.22 | 4.3 | 4.8 | 77.11 |
| F | 4.38 | 4.45 | 4.83 | 84.12 |
| Fen | 4.46 | 4.52 | 4.85 | 87.3 |
| Ant | 4.54 | 4.59 | 4.86 | 90.21 |
| Flu | 5.2 | 5.08 | 4.92 | 101.85 |
| Pir | 5.3 | 5.15 | 4.92 | 101.55 |
| BaA | 5.91 | 5.46 | 4.86 | 89.93 |
| Cri | 5.61 | 5.32 | 4.9 | 97.51 |
| BbF | 5.78 | 5.4 | 4.88 | 93.58 |
| BkF | 6.2 | 5.56 | 4.81 | 80.37 |
| BaP | 6.35 | 5.6 | 4.78 | 74.94 |
| Ind | 6.75 | 5.68 | 4.69 | 60.14 |
| DahA | 6.51 | 5.64 | 4.75 | 69.01 |
| BghiP | 6.9 | 5.7 | 4.65 | 54.78 |

Table S4. Estimated concentrations of PAH (Cw) in the reservoir stations (ng/L) (Mean ± Standard error)

| HAP | TSB  (n=4) | TPE  (n=4) | EES  (n=4) | EBT  (n=4) | ESB  (n=4) | EPA  (n=4) |
| --- | --- | --- | --- | --- | --- | --- |
| N | 250.9±213.4 | 205.3±63.74 | 430.7±232.7 | 917.8±703.3 | 230.13±80.64 | 237.95±77.24 |
| Acy | 1.02 ± 0.849 | 2.22±0.792 | 11.34±9.99 | 0.95±0.94 | 14.73±12.85 | 1.48±0.875 |
| Ace | --- | --- | ---- | ---- | ---- | ---- |
| F | 1.06 ± 0.634 | 1.71±0.609 | 2.78±1.85 | 7.54±3.70 | 1.91±0.395 | 4.29±1.76 |
| Fen | 8.02 ± 2.93 | 8.08±0.964 | 18.5±8.06 | 59.4±35.6 | 17.55±6.89 | 36.8±25.9 |
| Ant | 0.13 ± 0.075 | 0.176±0.168 | 1.87±0.865 | 2.34±0.769 | 0.409±0.260 | 1.68±1.26 |
| Flu | 0.57±0.29 | 0.675±0.246 | 1.47±0.634 | 4.64±3.08 | 1.00±0.357 | 6.76±6.01 |
| Pyr | 0.38±0.19 | 0.593±0.299 | 1.28±0.588 | 5.42±3.87 | 1.65±0.580 | 7.58±6.97 |
| BaA | ---- | ---- | ---- | --- | ---- | --- |
| Chr | 0.067±0.051 | 0.167±0.133 | 0.264±0.145 | 2.64±2.12 | 0.238±0.221 | 2.57±2.45 |
| BbF | 0.023±0.015 | 0.039±0.029 | 0.058±0.036 | 0.60±0.42 | 0.102±0.066 | 0.665±0.61 |
| BkF | 0.012±0.006 | 0.019±0.008 | 0.020±0.036 | 0.008±0.007 | 0.028±0.011 | 0.020±0.007 |
| BaP | 0.075±0.023 | 0.188±0.107 | 0.035±0.024 | 0.153±0.043 | 0.173±0.077 | 0.138±0.101 |
| Ind | 0.024±0.015 | 0.027±0.010 | 0.057±0.028 | 0.239±0.167 | 0.059±0.039 | 0.188±0.136 |
| DahA | 0.043±0.009 | 0.039±0.014 | < 0.002 | 0.081±0.079 | 0.027±0.015 | 0.108±0.067 |
| BghiP | 0.055±0.021 | 0.067±0.029 | 0.091±0.020 | 0.501±0.320 | 0.112±0.053 | 0.283±0.228 |
| ∑HAPs | 262.44 | 219.28 | 468.58 | 1002.38 | 268.14 | 300.58 |

Tabla S5. Principal components of PAHs from dialyzed extracts of SPMD.

| Variable | PC1 | PC2 | PC3 |
| --- | --- | --- | --- |
| N | 0.493 | **-0.763** | 0.354 |
| Acy | -0.116 | 0.465 | **0.602** |
| F | **0.800** | -0.421 | 0.147 |
| Phe | **0.784** | **0.540** | -0.0204 |
| Ant | **0.853** | 0.0518 | 0.066 |
| Flu | **0.881** | 0.416 | -0.169 |
| Pyr | **0.937** | 0.254 | -0.166 |
| Chr | **0.979** | -0.137 | -0.0161 |
| BbF | **0.991** | 0.0251 | -0.0752 |
| BkF | -0.185 | 0.379 | **0.691** |
| BaP | **0.772** | 0.232 | 0.297 |
| Ind | **0.939** | -0.262 | 0.110 |
| DahA | **0.792** | -0.430 | 0.147 |
| BghiP | **0.900** | 0.313 | -0.210 |
| Eigenvalue | 8.79 | 2.08 | 1.22 |
| Percentage of variance (%) | 62.79 | 14.85 | 8.73 |
| Cumulative Percentage (%) | 62.79 | 77.64 | 86.37 |

Tabla S6. values of toxicity NOEC (µg/L) of phenantrene exposure on ecological groups

| Taxa | Phylum | Class | Order | Species | Effect | Value (μg/L) NOEC |
| --- | --- | --- | --- | --- | --- | --- |
| Zooplankton |  |  |  | *Daphnia magna* | Production | 57 |
|  |  | Branchiopoda | Cladocera |  |  |  |
|  |  |  |  | *Daphnia pulex* | Reproduction | 60 |
|  |  |  | Mysida | *Neomysis awatschensis* | DNA damage | 25 |
|  |  |  | Amphipoda | *Corophium acherusicum* | DNA damage | 30 |
|  |  | [Malacostraca](https://en.wikipedia.org/wiki/Malacostraca) | Amphipoda | *Hyalella azteca* | Mortality | 10000 |
|  | Arthropoda |  |  |  |  |  |
|  |  |  | [Harpacticoida](https://en.wikipedia.org/wiki/Harpacticoida) | *Tigriopus japonicus* | Reproduction | 10 |
|  |  | [Hexanauplia](https://en.wikipedia.org/wiki/Hexanauplia) | [Harpacticoida](https://en.wikipedia.org/wiki/Harpacticoida) | *Tigriopus fulvus* | Mortality | 200 |
|  |  |  |  |  |  |  |
|  |  |  |  |  |  |  |
|  | Mollusca |  | [Mytiloida](https://es.wikipedia.org/wiki/Mytiloida) | *Mytilus edulis* | Enzyme | 50 |
|  |  | Bivalvia | [Mytiloida](https://es.wikipedia.org/wiki/Mytiloida) | *Mytilus galloprovincialis* | Cell | 100 |
|  |  |  |  |  |  |  |
| Microalgae | Bacillariophyta | Coscinodiscophyceae | [Thalassiosirales](https://en.wikipedia.org/wiki/Thalassiosirales) | *Thalassiosira pseudonana* | Growth inhibition | 125 |
|  |  |  | [Sphaeropleales](https://en.wikipedia.org/wiki/Sphaeropleales) | *Scenedesmus subpicatus* | Population | 2740 |
|  | Chlorophyta | Chlorophyceae | [Sphaeropleales](https://en.wikipedia.org/wiki/Sphaeropleales) | *Scenedesmus armatus* | Population | 5000 |
|  |  | Ulvophyacea | Ulvales | *Enteromorpha linza* | Growth inhibition | 0,988 |
| *FISH* |  |  | Salmoniformes | *Oncorhynchus mykiss* | Mortality | 66 |
|  |  |  | [Pleuronectiformes](https://es.wikipedia.org/wiki/Pleuronectiformes) | *Paralichthys olivaceus* | Enzyme | 89,1 |
|  |  |  |  | *Oryzias latipes* | Development | 100 |
|  | Chordata | [Actinopterygii](https://es.wikipedia.org/wiki/Actinopterygii) | Beloniformes | *Oryzias melastigma* | Hatching | 50 |
|  |  |  | [Perciformes](https://www.google.com/search?q=Perciformes&stick=H4sIAAAAAAAAAOPgE-LUz9U3sEwpz7BQ4gAxTctN0rR0M8qt9JPzc3JSk0sy8_P084vSE_Myi3Pjk3MSi4sz0zKTE0HixVb5RSmpRYtYuQNSi5Iz0_KLclOLd7AyAgBq900xVwAAAA&sa=X&ved=2ahUKEwjhptOY8dDrAhVQnlkKHbJEBjwQmxMoATAaegQIDxAD&biw=1366&bih=654) | *Sparus macrocephalus* | Hatching | 50 |
|  |  |  | Mugiliformes | *Liza aurata* |  | 481 |
| Macrophytas | Magnoliophyta | Liliopsida | Alismatales | *Lemna minor* | Population | 658 |
| *BENTHIC INVERTEBRADES* | Annelida | Clitellata | [Lumbriculida](https://en.wikipedia.org/wiki/Lumbriculida) | *Lumbriculus variegatus* |  | 419 |
|  |  |  |  |  |  |  |
|  |  | [Polychaeta](http://www.marinespecies.org/aphia.php?p=taxdetails&id=883) | [Phyllodocida](http://www.marinespecies.org/aphia.php?p=taxdetails&id=892) | *Neanthes arenaceodentata* |  | 669 |
|  |  |  |  |  |  |  |

Tabla S7 . values of toxicity NOEC (µg/L) of fluoranthene exposure on ecological groups

| Taxa | Phylum | Class | Order | Scientific name | Effect | Value (μg/L) NOEC |
| --- | --- | --- | --- | --- | --- | --- |
| MICROALGAE | [Chlorophyta](https://www.algaebase.org/browse/taxonomy/?id=97241) | [Chlorophyceae](https://en.wikipedia.org/wiki/Chlorophyceae) | [Sphaeropleales](https://en.wikipedia.org/wiki/Sphaeropleales) | *Ankistrodesmus sp,* | Biochemistry | 19 |
|  | [Chlorophyta](https://www.algaebase.org/browse/taxonomy/?id=97241) | [Trebouxiophyceaee](https://www.algaebase.org/browse/taxonomy/?id=4356) | [Chlorellales](https://www.algaebase.org/browse/taxonomy/?id=4515) | *Chlorella fusca var, vacuolata* | Population | 12,74 |
|  | [Chlorophyta](https://www.algaebase.org/browse/taxonomy/?id=97241) | [Chlorophyceae](https://en.wikipedia.org/wiki/Chlorophyceae) | [Sphaeropleales](https://en.wikipedia.org/wiki/Sphaeropleales) | *Pseudokirchneriella subcapitata* | Population | 41,7 |
|  | [Bacillariophyta](https://www.algaebase.org/browse/taxonomy/?id=139141) | Bacillariophycea | [Bacillariales](https://en.wikipedia.org/wiki/Bacillariales) | *Phaeodactylum tricornutum* | Enzyme(s) | 100 |
| AMPHIBIA | [Chordata](https://es.wikipedia.org/wiki/Chordata) | [Amphibia](https://es.wikipedia.org/wiki/Amphibia) | [Anura](https://es.wikipedia.org/wiki/Anura) | *Rana catesbeiana* | Behavior | 10,97 |
| ZOOPLANCTON |  | Malacostraca | Amphipoda | *Diporeia sp,* | Mortality | 861,6 |
|  |  | Cladocera |  | *Daphnia magna* | Mortality | 85 |
|  |  | Malacostraca | Mysida | *Americamysis bahia* | Mortality | 21 |
|  |  | Malacostraca | Amphipoda | *Hyalella azteca* | Feeding behavior | 12,5 |
|  | Arthropoda | Malacostraca | Amphipoda | *Leptocheirus plumulosus* | Growth | 212 |
|  |  | Malacostraca | Amphipoda | *Ampelisca abdita* | Biochemistry | 3,5 |
|  |  | Malacostraca | Amphipoda | *Rhepoxynius abronius* | Biochemistry | 3,5 |
|  |  | Malacostraca | Decapoda | *Palaemonetes pugio* | Mortality | 22 |
|  |  | Insecta | [Diptera](https://es.wikipedia.org/wiki/Diptera) | *Aedes aegypti* | Genetics | 250 |
|  |  | Insecta | [Diptera](https://es.wikipedia.org/wiki/Diptera) | *Chironomus riparius* | Development | 43 |
|  |  | Insecta | [Diptera](https://es.wikipedia.org/wiki/Diptera) | *Chironomus tentans* | Mortality | 20 |
| *FISH* | Chordata | [Actinopterygii](https://es.wikipedia.org/wiki/Actinopterygii) | Cypriniformes | *Pimephales promelas* | Mortality | 10,4 |
|  |  |  |  | *Danio rerio* | Histology | 500 |
| *MACROPHYTA* |  |  |  | *Plantae* | Biochemistry | 95 |
|  | Magnoliophyta | Liliopsida | Alismatales | *Lemna minor* | Population | 166 |
| *BENTHIC INVERTEBRADES* | Annelida | [Oligochaeta](http://animalandia.educa.madrid.org/ficha-taxonomica.php?id=4161&nivel=Clase&nombre=Oligochaeta) | [Haplotaxida](http://animalandia.educa.madrid.org/ficha-taxonomica.php?id=4161&nivel=Orden&nombre=Haplotaxida) | *Stylaria lacustris* | Mortality | 115 |
|  | Mollusca | Bivalvia | [Veneroida](https://es.wikipedia.org/wiki/Veneroida) | *Ruditapes decussatus* | Mortality | 88 |
|  |  |  | [Ostreida](https://es.wikipedia.org/w/index.php?title=Ostreida&action=edit&redlink=1) | *Crassostrea virginica* | Enzyme(s) | 10 |
|  |  |  | [Cardiida](https://en.wikipedia.org/w/index.php?title=Cardiida&action=edit&redlink=1) | *Macomona liliana* | Behavior | 50 |

Tabla S8. values of toxicity NOEC (µg/L) of Benzo(a)pyrene exposure on ecological groups

| TAXA | Phylum | Class | Order | Scientific name | Effect | Value (μg/L) NOEC |
| --- | --- | --- | --- | --- | --- | --- |
| Zooplankton | Arthropoda | Insecta | [Diptera](https://es.wikipedia.org/wiki/Diptera) | *Aedes aegypti* | Enzyme | 5 |
|  |  |  |  |  |  |  |
|  |  | Insecta | [Diptera](https://es.wikipedia.org/wiki/Diptera) | *Chironomus tentans* | Growth inhibition | 500 |
|  |  |  |  |  |  |  |
|  |  | Insecta | [Diptera](https://es.wikipedia.org/wiki/Diptera) |  | Genetic | 100 |
|  |  |  |  | *Chironomus riparius* |  |  |
|  |  | Branchiopoda | Cladocera | *Daphnia magna* |  | 0,02 |
|  |  |  |  |  |  |  |
|  |  | [Hexanauplia](https://en.wikipedia.org/wiki/Hexanauplia) | [Calanoida](http://www.marinespecies.org/aphia.php?p=taxdetails&id=1100) | *Eurytemora affinis* | Development | 12 |
|  |  | [Hexanauplia](https://en.wikipedia.org/wiki/Hexanauplia) | [Harpacticoida](https://en.wikipedia.org/wiki/Harpacticoida) | *Tigriopus japonicus* | Reproduction | 1,8 |
| *Protozooa* | Ciliophora | [Oligohymenophorea](https://en.wikipedia.org/wiki/Oligohymenophorea) | [Peniculida](https://en.wikipedia.org/wiki/Peniculida) | Paramecium caudatum | Intoxication | 1000 |
|  |  |  |  |  |  |  |
|  |  | [Oligohymenophorea](https://en.wikipedia.org/wiki/Oligohymenophorea) | [Peniculida](https://en.wikipedia.org/wiki/Peniculida) | *Paramecium aurelia* | Intoxication | 10 |
| *MICROALGAE* |  | [Bacillariophyceae](https://www.algaebase.org/browse/taxonomy/?id=4337) | [Bacillariales](https://en.wikipedia.org/wiki/Bacillariaceae) | *Nitzscnia closterium* | Growth inhibition | 0,15 |
|  | Bacillariophyta | [Mediophyceae](https://www.algaebase.org/browse/taxonomy/?id=139117) | [Thalassiosirales](https://www.algaebase.org/browse/taxonomy/?id=4465) | *Skeletonema costatum* | Growth inhibition | 0,17 |
|  |  | Coscinodiscophyceae | [Thalassiosirales](https://en.wikipedia.org/wiki/Thalassiosirales) | *Thalassiosira pseudonana* | Growth inhibition | 0,36 |
|  | Chlorophyta | Ulvophyacea | Ulvales | *Enteromorpha linza* | Growth inhibition | 160 |
| FISH | Chordata |  | Beloniformes | *Oryzias melastigma* | Hatching | 10 |
|  |  |  | Cypriniformes | *Cyprinus carpio* | Genetic | 0,86 |
|  |  |  | Cypriniformes | *Carassius auratus* | Enzyme | 0,3 |
|  |  | [Actinopterygii](https://es.wikipedia.org/wiki/Actinopterygii) | [Cypriniformes](https://es.wikipedia.org/wiki/Cypriniformes) | *Zacco platypus* |  | 24 |
|  |  |  |  |  |  |  |
|  |  |  | Mugiliformes | *Mugil soiuy* | Enzyme | 0,1 |
|  |  |  | [Scorpaeniformes](https://en.wikipedia.org/wiki/Scorpaeniformes) | *Sebastiscus marmoratus* | Enzyme | 0,01 |
|  |  |  | [Clupeiformes](https://en.wikipedia.org/wiki/Clupeiformes) | *Dorosoma cepedianum* |  | 1,01 |
|  |  |  |  |  |  |  |
|  |  |  | [Siluriformes](https://es.wikipedia.org/wiki/Siluriformes) | *Clarias gariepinus* |  | 54,7 |
|  |  |  |  |  |  |  |
| BENTHIC INVERTEBRADES | Mollusca | Bivalvia | [Mytiloida](https://es.wikipedia.org/wiki/Mytiloida) | *Mytilus galloprovincialis* | Genetic | 100 |
|  |  | Gastropoda | [Basommatophora](https://es.wikipedia.org/wiki/Basommatophora) | Physella acuta | Mortality | 20 |
|  |  |  |  |  |  |  |
|  | [Chordata](https://es.wikipedia.org/wiki/Chordata) | [Amphibia](https://es.wikipedia.org/wiki/Amphibia) | [Anura](https://es.wikipedia.org/wiki/Anura) | *Xenopus laevis* |  | 33,3 |
|  | [Ciliophora](http://www.marinespecies.org/aphia.php?p=taxdetails&id=11) | [Spirotrichea](http://www.marinespecies.org/aphia.php?p=taxdetails&id=1348) | [Euplotida](http://www.marinespecies.org/aphia.php?p=taxdetails&id=341297) | Euplotes crassus | Physiology | 12 |
|  |  |  |  |  |  |  |
|  | Mollusca | Bivalvia | [Pectinida](https://en.wikipedia.org/wiki/Pectinida) | Chlamys farreri | Hystology | 2,93 |
|  |  |  |  |  |  |  |
| Chell | [Chordata](https://es.wikipedia.org/wiki/Chordata) | [Reptilia](https://es.wikipedia.org/wiki/Reptilia) | [Testudines](https://es.wikipedia.org/wiki/Testudines) | Caretta caretta | Genetics | 2,53 |
